# Second Order Dimensionality Reduction Using Minimum and Maximum Mutual Information Models

**DOI:** 10.1371/journal.pcbi.1002249

**Published:** 2011-10-27

**Authors:** Jeffrey D. Fitzgerald, Ryan J. Rowekamp, Lawrence C. Sincich, Tatyana O. Sharpee

**Affiliations:** 1 Computational Neurobiology Laboratory, The Salk Institute for Biological Studies, La Jolla, California, United States of America; 2 Center for Theoretical Biological Physics and Department of Physics, University of California, San Diego, California, United States of America; 3 Department of Vision Sciences, University of Alabama at Birmingham, Birmingham, Alabama, United States of America; Indiana University, United States of America

## Abstract

Conventional methods used to characterize multidimensional neural feature selectivity, such as spike-triggered covariance (STC) or maximally informative dimensions (MID), are limited to Gaussian stimuli or are only able to identify a small number of features due to the curse of dimensionality. To overcome these issues, we propose two new dimensionality reduction methods that use minimum and maximum information models. These methods are information theoretic extensions of STC that can be used with non-Gaussian stimulus distributions to find relevant linear subspaces of arbitrary dimensionality. We compare these new methods to the conventional methods in two ways: with biologically-inspired simulated neurons responding to natural images and with recordings from macaque retinal and thalamic cells responding to naturalistic time-varying stimuli. With non-Gaussian stimuli, the minimum and maximum information methods significantly outperform STC in all cases, whereas MID performs best in the regime of low dimensional feature spaces.

## Author Summary

Neurons are capable of simultaneously encoding information about multiple features of sensory stimuli in their spikes. The dimensionality reduction methods that currently exist to extract those relevant features are either biased for non-Gaussian stimuli or fall victim to the curse of dimensionality. In this paper we introduce two information theoretic extensions of the spike-triggered covariance method. These new methods use the concepts of minimum and maximum mutual information to identify the stimulus features encoded in the spikes of a neuron. Using simulated and experimental neural data, these methods are shown to perform well both in situations where conventional approaches are appropriate and where they fail. These new techniques should improve the characterization of neural feature selectivity in areas of the brain where the application of currently available approaches is restricted.

## Introduction

In recent years it has become apparent that many types of sensory neurons simultaneously encode information about more than one stimulus feature in their spiking activity. Examples can be found across a wide variety of modalities, including the visual [1]–[12], auditory [13], olfactory [14], somatosensory [15] and mechanosensory [16] systems. This discovery was facilitated by the development of dimensionality reduction techniques like spike-triggered covariance (STC) [17]–[22] and maximally informative dimensions (MID) [23]. These two methods exhibit complementary advantages and disadvantages. For instance, STC can identify many relevant features for stimuli whose parameters are distributed in a Gaussian manner but can fail when natural stimuli are used, whereas MID works well for arbitrary stimuli but requires exponentially larger data sets to find more than a few features. Therefore, there is need for a method that can find relevant features from arbitrary stimulus distributions while bypassing the curse of dimensionality. Here we propose two novel techniques based on minimum and maximum mutual information; these new approaches can be seen as an extension of STC to arbitrary stimuli.

Neural coding of multiple stimulus features is typically modeled as a linear-nonlinear Poisson (LNP) process [24]–[28]. A stimulus 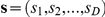, such as an image with 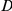 pixels, as well as each of the 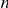 features 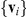 for which a neuron is selective are represented by vectors in a 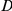 dimensional space. The neuron extracts information about the stimulus by projecting 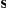 onto the linear subspace spanned by the feature vectors. The result is a stimulus of reduced dimensionality 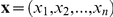, with 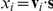; this input is then passed through an nonlinear firing rate function 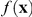. Spikes are then assumed to be generated by a Poisson process with a rate equal to 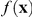, which only depends on the relevant dimensions of the stimulus space.

Given a set of stimuli 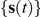, for 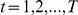 and the corresponding observed neural responses 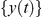, where 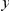 is number of spikes, there are a few commonly used methods available to extract the stimulus features relevant to the neuron. In the STC method, the stimulus covariance matrix 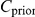 and the covariance of the spike-triggered ensemble,
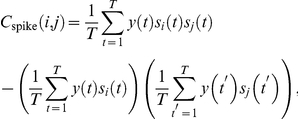
 are compared to discover the dimensions along which the stimulus variance conditional on a spike is significantly different from the stimulus variance overall. This comparison is done by diagonalizing the matrix 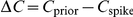. The relevant features can be identified by the eigenvectors that have nonzero eigenvalues. If the stimuli are drawn from a distribution 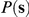 which is Gaussian, then the only limitation to finding the features is having a large enough set of spike data. In practice, the STC procedure can be extended to Gaussian stimuli containing correlations by adding a whitening step [17], [18], and can also include a regularization term to smooth the results (see Methods). On the other hand, if 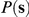 is non-Gaussian, as is the case for natural images, then higher order stimulus correlations can greatly affect the results [23], 29.

The use of Gaussian stimuli makes it possible to find many relevant dimensions using STC, but fully sampling the dynamic range of responses often requires a 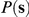 more similar to the non-Gaussian distributions found in nature [27], [30]. It has also been suggested that neural representations of stimuli may be optimized in some way [31]–[33] to the statistics of the natural environment. With this in mind, it is important that multidimensional feature extraction methods be extended to stimulus distributions with non-Gaussian statistics.

The MID method is an information theoretic dimensionality reduction technique that identifies relevant features based on how much information a linear subspace contains about the observed spikes (see Methods). Unlike STC, the dimensionality of the relevant subspace to be found using MID must be specified *a priori*, and thus to discover the number of relevant features one must search for additional dimensions until the subspace accounts for a sufficient fraction of the information carried in the neural response. The objective function in MID relies on an empirical construction of the reduced stimulus distribution 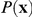 and the corresponding conditional distribution 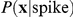, and thus suffers from the curse of dimensionality [34]. A related problem that occurs equally for Gaussian and non-Gaussian stimuli, and affects both the STC and MID methods, is that even if one is able to find many relevant dimensions, it is usually not possible to sample the nonlinear gain function simultaneously along all of these dimensions.

Here we put forth two new dimensionality reduction techniques applicable to arbitrary stimulus distributions. These methods, much like STC, make use of pairwise correlations between stimulus dimensions and are not hindered by the curse of dimensionality in the same manner as MID. To demonstrate the usefulness of the proposed methods, we apply them to simulated neural data for two biologically inspired model cells, and to physiological recordings of the response of macaque retina and thalamus cells to time-varying stimuli.

## Results

### Dimensionality reduction using minimal models

If the spiking activity of a neuron is encoding certain aspects of the stimulus, then the corresponding stimulus features must be correlated in some way with the neural response. From an experiment one can estimate specific stimulus/response correlations, such as the spike-triggered average (STA), the spike-triggered covariance (STC), or the mutual information [35],
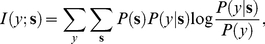
(1)which provides a full measure of the degree of dependence between stimulus and response. These estimates can then be used to construct a model of the conditional response probability by constraining 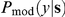 to match a given set of observed correlations, as in the STA and STC methods. As there are an infinite number of models that match any given set of experimentally estimated correlations, the values of the unconstrained correlations are necessarily determined by the specific choice of 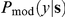.

The minimal model of 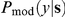 is the one that is consistent with the chosen set of correlations but is otherwise as random as possible, making it minimally biased with respect to unconstrained correlations [36]. This model can be obtained by maximizing the noise entropy 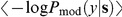, where 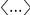 denotes an average over 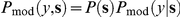. For a binary spike/no spike neuron consistent with an observed mean firing rate, as well as the correlation of the neural response with linear and quadratic moments of the stimulus, the minimal model is a logistic function [36]

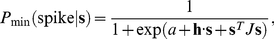
(2)where the parameters 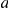, 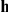 and 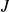 are chosen such that the mean firing rate, STA and STC of the model match the experimentally observed values (see Methods). If correlations between a spike and higher order moments of the stimulus are measured, the argument of the logistic function would include higher powers of 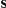. In addition to being as unbiased as possible, 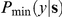 also minimizes the mutual information [36], [37], which only includes the contribution of the chosen constraints. We note that previously we used this minimal model framework to characterize the computation performed within the reduced relevant subspace [36], and in particular to quantify in information-theoretic terms the contribution of higher-than-second powers of relevant stimulus features to neural firing. Here, we study whether analysis of the second-order minimal models constructed in the full stimulus space can be used to find the relevant feature subspace itself.

**Figure 1:**
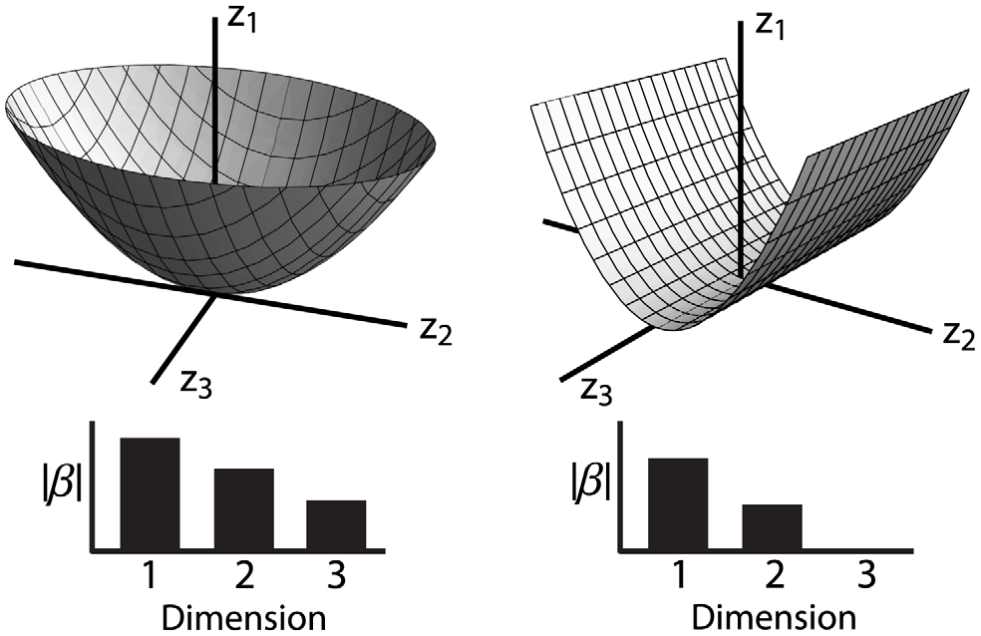
Eigenvector analysis of quadratic probability surfaces. The 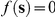 surfaces are shown for two simple second order minimal models in a three dimensional space. For the surface on the left all three eigenvalues are nonzero; the surface curves in all three dimensions and the neuron is selective for three features. For the surface on the right one of the eigenvalues is equal to zero; the surface only curves in two dimensions and the neuron is selective for only two features.

The contours of constant probability of the minimal second order models are quadric surfaces, defined by the quadratic polynomial 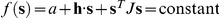. The diagonalization of 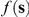 involves a change of coordinates such that
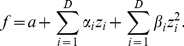
(3)This is accomplished through the diagonalization of the matrix 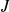, yielding 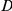 eigenvectors 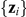 with corresponding eigenvalues 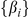. These eigenvectors are the principal axes of the constant probability surfaces, and as such the magnitude of the eigenvalue along a particular direction is indicative of the curvature, and hence the selectivity, of the surface in that dimension. This point is illustrated in Fig. 1.

The linear term in Eq. (3) may also contain a significant feature. Subtracting off the relevant dimensions found from diagonalizing 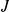 leaves an orthogonal vector 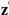. The magnitude of this vector can be directly compared to the eigenvalue spectrum to determine its relative strength.

### Dimensionality reduction using nonlinear MID

The minimal models of binary response systems take the form of logistic functions. This restriction can be eliminated if we look for a maximally informative second order model. To accomplish this, we extend the MID algorithm to second order in the stimulus by assuming the firing rate is a function of a quadratic polynomial, 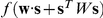. The nonlinear MID (nMID) algorithm is then run exactly as linear MID in the expanded 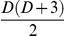 dimensional space.

Once the maximally informative parameters are found, the matrix 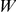 can be diagonalized to reveal the relevant features, and the linear term can be analyzed in the same manner as for the minimal sigmoidal model. The ability to construct an arbitrary nonlinearity allows nonlinear MID to include information contained in higher order stimulus/response correlations and to find the linear combination that captures the most information about the neural response. Unlike multidimensional linear MID, nonlinear MID is one-dimensional in the quadratic stimulus space and therefore avoids the curse of dimensionality in the calculation of the objective function.

### Application to simulated neurons

To test and compare the two proposed methods, both to each other and to the established methods such as STC and MID, we created two model cells designed to mimic properties of neurons in primary visual cortex (V1). The first model cell was designed to have two relevant dimensions, which places it in the regime where the linear MID method should work. The second model was designed to have six relevant dimensions and serves as an example of a case that would be difficult to characterize with linear MID. Using the van Hateren [38] natural image database, a different set of 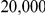 patches of 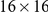 pixels were randomly selected as stimuli for each cell; 100 repetitions of these image sequences were presented during the course of the simulated experiment.

To quantify the performance of a given dimensionality reduction method, we calculate the subspace projection [39]

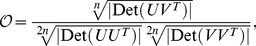
(4)where 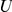 is an 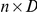 matrix whose rows are the 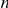 most significant dimensions found from either 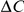, 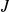 or 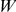, and 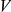 is a matrix containing the 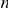 model cell features. This quantity is the intersection of the volumes spanned by the two sets of vectors. It is bounded between 0 and 1, with 0 meaning the two subspaces have no overlap and 1 meaning they are identical, and is invariant to a change of basis or rescaling of the vectors in either subspace.

**Figure 2:**
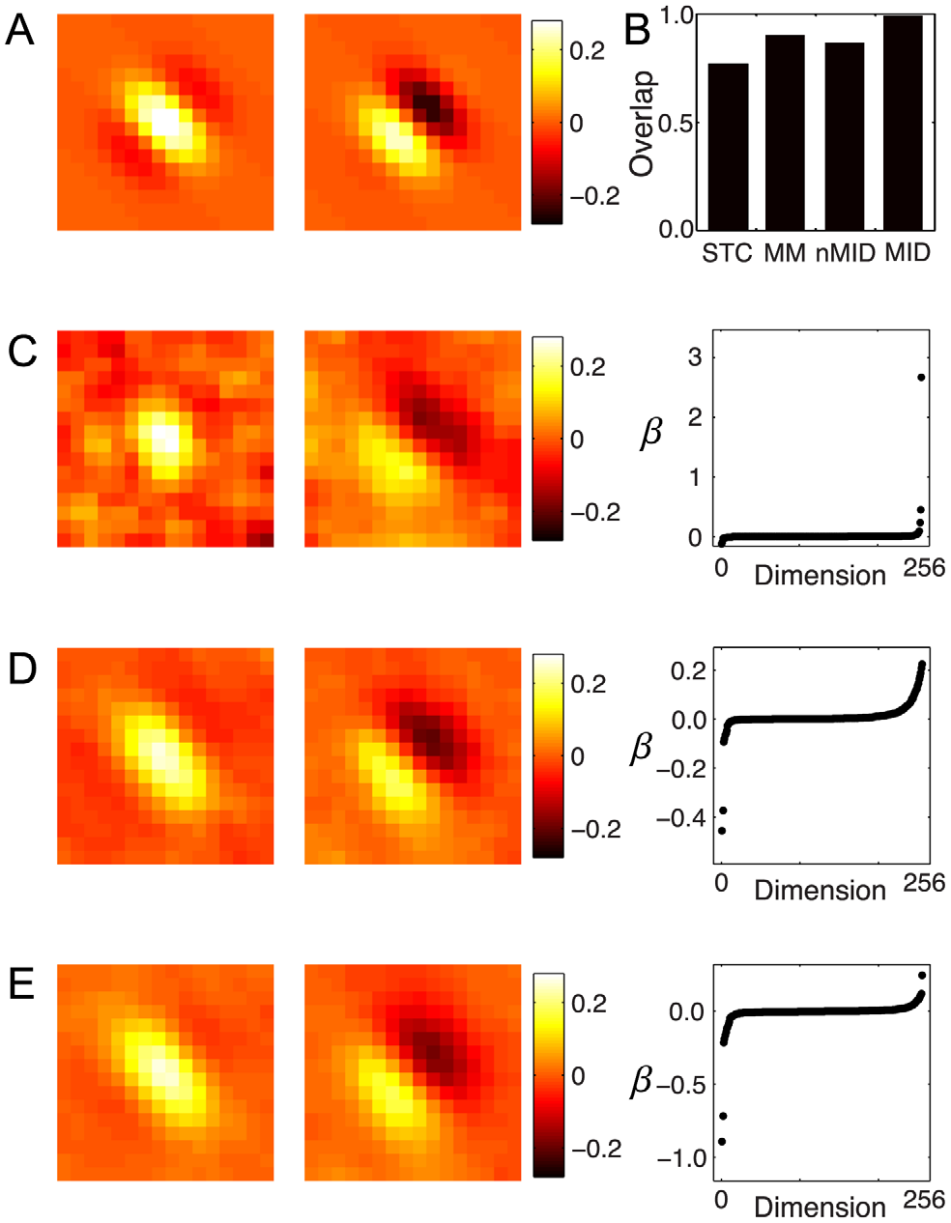
Model complex cell. **A**) The two excitatory features of the model are Gabor filters 90 degrees out of phase. The quadratic nonlinearity ensures that the responses are invariant to phase. **B**) Subspace projections for the STC, minimal model (MM), and nonlinear and linear MID models. The normalized eigenvectors (left) corresponding to the two largest magnitude eigenvalues (right) for **C**) STC, **D**) minimal model and **E**) nonlinear MID method.

The first model cell was constructed to respond to the two Gabor features shown in Fig. 2A in a phase invariant manner. This cell approximates a complex cell in area V1 by responding to the square of the stimulus projections onto the Gabor features, with a firing rate proportional to 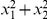, as in the energy model [7], [40]–[45]. Although the firing rate was low for this model cell, there was occasionally more than one spike per stimulus frame. These instances were rare and to simplify the analysis the neural response was binarized by setting all multiple spiking events equal to one.

As expected, the STC method performed poorly due to the strong non-Gaussian properties of natural stimuli [30], [46]. The STC method found a subspace with an overlap of 0.77, whereas the nonlinear MID result had an overlap of 0.87 and the minimal model subspace had an overlap of 0.90, as shown in Fig. 2B. For comparison, the conventional MID method searched for the two most informative dimensions and was able to recover a subspace that almost perfectly reproduced the ground truth, with an overlap of 0.98. The feature vectors found by the different methods and the corresponding eigenvalue spectra are shown in Fig. 2C–E.

**Figure 3:**
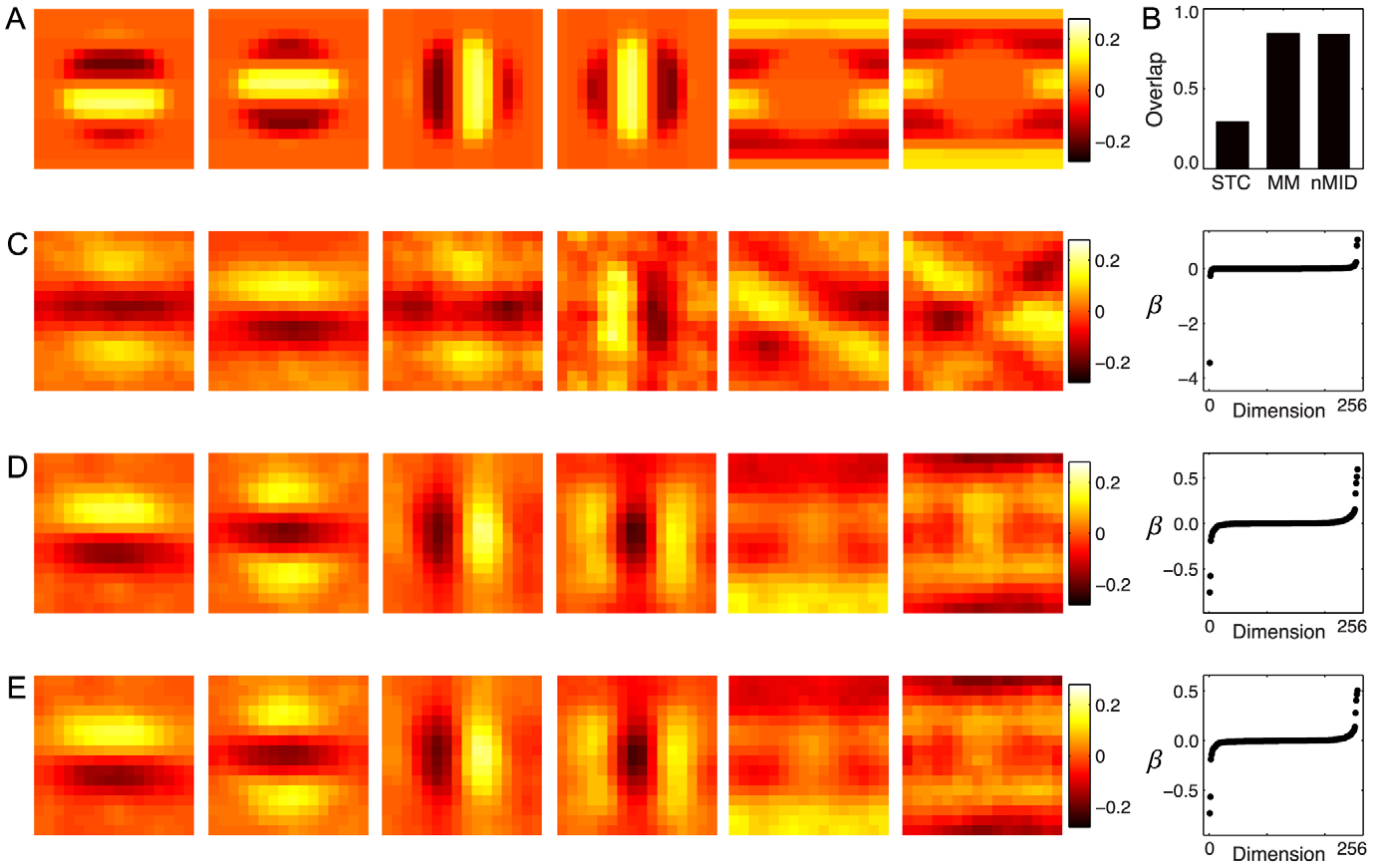
Model complex cell with inhibitory features. **A**) The first two panels show the excitatory fields: two Gabor filters 90 degrees out of phase located only in the center region of the receptive field (RF). The middle two panels show two inhibitory Gabor features, also in the middle of the RF and rotated to have an orientation perpendicular to that of the excitatory features. The right two panels show two inhibitory surround features aligned in orientation to the excitatory features. A quadratic nonlinearity applied to the projection of the stimulus onto these six features ensures phase invariance. **B**) The subspace projections for the STC, minimal model (MM) and nonlinear MID models. The eigenvectors (left) corresponding to the six largest magnitude eigenvalues (right) using the **C**) STC, **D**) minimal models and **E**) nonlinear MID method.

A second model cell was also created to resemble a V1 complex cell, but with a divisive normalization based on inhibitory features with orthogonal orientation in the center and parallel orientation in the surround [7], [40]–[45], [47], as shown in Fig. 3A. The two excitatory features in the center of the receptive field have a specific orientation. The two inhibitory features in the center of the receptive field have an orientation orthogonal to that of the excitatory features, while the two suppressive features in the surround have the same orientation as the excitatory ones in the center. The nonlinear gain function for this cell is
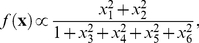
(5)scaled such that the average spike probability over the stimulus set was approximately 0.15. Spiking responses were binarized as for the first model cell.

The performance of the various dimensionality reduction methods is shown in Fig. 3B. The spike-triggered covariance approach finds features (Fig. 3C) that bear some resemblance to the model features, but have a low overlap of 0.29. In contrast, nonlinear MID and the minimal model find features with much larger overlaps: 0.84 and 0.85, respectively. Note that the linear MID was not implemented for this model cell, as the algorithm cannot recover a 6 dimensional feature space.

### Feature selectivity of real neurons

To demonstrate the usefulness of the new approaches proposed here for the analysis of real neural data, we analyzed the responses of 9 macaque retina ganglion cells (RGC) and 9 cells from the lateral geniculate nucleus (LGN) under naturalistic stimulus conditions [48] (see Methods). In this case, the stimulus was a spot of light filling the center of the RGC or LGN receptive field with non-Gaussian intensity fluctuations.

While we cannot know the true features of these neurons as we can for the model cells, this data was previously analyzed using MID [3] and it was found that two stimulus features explain nearly all of the information in the neural response (an average of 85% information explained across the 18 cells analyzed). We can therefore use the two linear MID features as a benchmark for comparing the features recovered with the new algorithms, using the subspace projection quantity in Eq. (4). Moreover, the veracity of these new algorithms can be tested by comparison with other studies that have used Gaussian stimuli and STC to investigate feature selectivity of retinal cells. For instance, it was previously shown that salamander RGCs are selective to 2 to 6 significant stimulus features [2]. Here we examine if the new algorithms can find a similar number of features in macaque RGCs.

**Figure 4:**
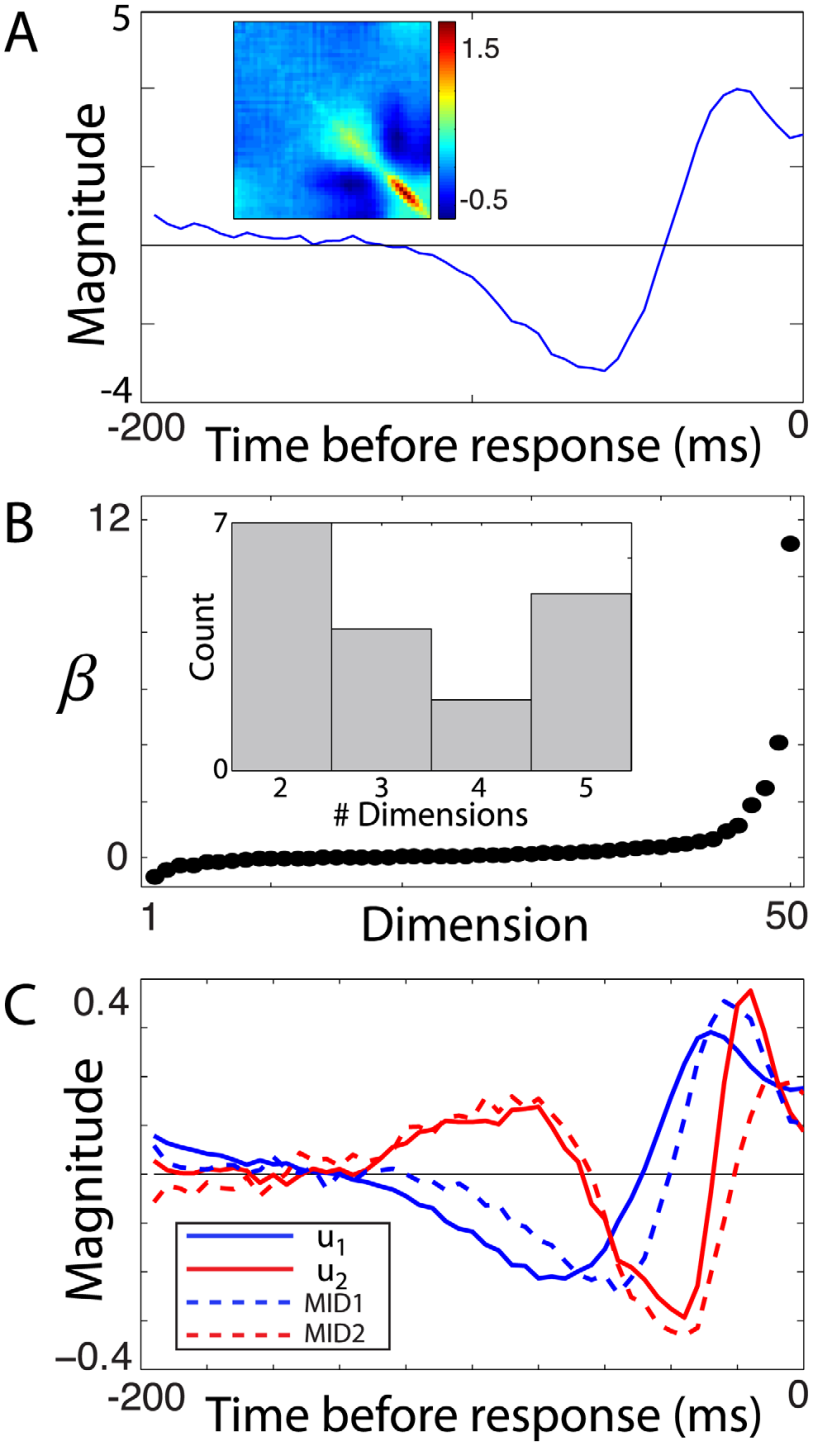
Minimal model of retinal feature selectivity in a retinal ganglion cell. A second order minimal model was fit to the spike train of a RGC. **A**) The feature 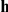 that controls the linear term in the argument of the logistic nonlinearity, plotted as a function of time before the neural response. The matrix 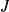 that controls the quadratic term is shown as an inset. **B**) The eigenvalue spectrum for this cell has two significant features. The inset shows a histogram of the number of significant features across the population of 9 retinal cells and 9 thalamic cells. All cells fell in the range of 2 to 5 features. **C**) The minimal model eigenvectors 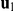 and 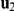 corresponding to the two largest eigenvalues (solid) along with the two most informative features (dashed). The most informative dimensions and these eigenvectors had a subspace projection of 0.93. This analysis thus validates the minimal model algorithm by applying it to neural data in a case where the relevant dimensions can be obtained by an existing and well established method.

We show the result of fitting the minimal model to one of the RGCs. The parameters are shown in Fig. 4A; the 50 dimensional linear term 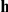 is plotted as a function of time before a spike and the matrix 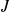 is shown in the inset. The eigenvalue spectrum of this cell is shown in Fig. 4B. The eigenvectors corresponding to the two largest eigenvalues are shown in Fig. 4C (solid curves); the MID features (dashed curves), shown for comparison, captured 92% of the information. These two subspaces are very similar, with an overlap of 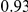, demonstrating that the minimal model method is able to accurately identify the two features of this cell.

Although the two most informative dimensions captured a very large percentage of the information in the neural response [3], the number of significant features found using the minimal model approach ranged from 2 to 5, echoing the previous work [2] in salamander retina using white noise stimuli and STC. The number of cells with a given number of significant features is shown in the histogram in Fig. 4B. Most of the cells were dominated by one or two features, with additional weakly influential dimensions having significant curvature, in agreement with previous findings [2], [3].

## Discussion

Both of the methods proposed here find relevant subspaces using second order stimulus statistics and can therefore be seen as extensions of the STC method. The minimal model is forced to have a logistic function nonlinearity, which has the benefit of removing unwanted model bias regarding higher than second order stimulus moments. In contrast, nonlinear MID uses an arbitrary nonlinear gain function and is therefore able to make use of higher order statistics to maximize information. Although both methods yield models consistent with first and second order stimulus/response correlations, neither method is guaranteed to work if the underlying neural computation does not match the structure of the model or the assumptions that underlie the estimation of relevant features.

In principle, the flexibility in the nonlinear MID gain function means it should perform at least as well as the minimal model. However, what we have observed is that the nonlinear MID subspace projection with these two model cells is slightly smaller than the minimal model subspace. This may be due to the differences in the nature of the optimization problems being solved in the two methods. Maximizing noise entropy under constraints is a convex optimization problem [49], whereas maximizing mutual information is not convex. This means that the parameter space for nonlinear MID may contain many local maxima. Although the MID algorithm uses simulated annealing to overcome this issue, the number of iterations required to outperform the minimal model may be large. We have observed (data not shown) that minimal models can find feature spaces with extremely high dimensionality 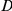, i.e. 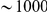, which corresponds to finding on the order of 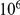 values of the covariance matrix.

Neurons with selectivity for only a few features that are probed with non-Gaussian stimuli, such as the model cell shown in Fig. 2 or the RGC in Fig. 4, can be characterized very well with MID, as previously shown [23]. Thus, in such cases MID is a useful tool for estimating the relevant features. We have found that for both real and model neurons with a small number of relevant features, the minimum and maximum information models performed quite well, despite the large number of parameters that need to be estimated. In particular, both methods were able to outperform STC in the recovery of the relevant stimulus subspace. On the other hand, when the dimensionality of the feature space is larger, as for the 6 dimensional cell in Fig. 3, linear MID cannot be used reliably due to the massive amount of data needed to construct a 6 dimensional empirical spike-conditional probability distribution. Because in the case of model cells the relevant features are known, we can verify that the minimal models and nonlinear MID approaches are able to find all of the features, whereas STC performs significantly worse. Furthermore, the fact that the second-order minimal models yielded a similar number (2–5) of relevant dimensions across the neural population as was previously described with Gaussian stimuli can be viewed as a further validation of the new method. It is our hope that these new techniques will advance the characterization of neural feature selectivity under a variety of stimulus conditions.

## Methods

### Ethics statement

Experimental data were collected as part of a previous study using procedures approved by the UCSF Institutional Animal Care and Use Committee, and in accordance with National Institutes of Health guidelines.

### Spike-triggered covariance

When applied to stimuli with correlations, a whitening procedure can be used to correct for them [18]. This procedure can still be used if stimuli are non-Gaussian, but the results are biased [29]. The whitening operation can be performed after diagonalization of 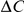 by multiplying the eigenvectors by 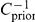, the inverse of the prior covariance matrix.

Whitening has the consequence of amplifying noise along poorly sampled dimensions. To combat this effect, we regularize using a technique called ridge regression [50] in our analysis, in which 
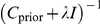
 instead of 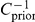 is used in the whitening step. Here 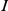 is the identity matrix and 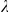 is a regularization parameter that was varied for both model cells to identify the value which gave the largest overlap. This value of 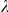 was used to give a best case estimate of STC performance. We note that this procedure gives more credit to STC compared to the other methods used here because it is not possible to evaluate a cross-validation metric such as percent information explained when many dimensions are involved.

### Maximally informative dimensions

Maximally informative dimensions [23] is an algorithm that finds one or more linear combinations of the stimulus dimensions, i.e. a reduced stimulus vector 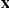, that maximizes the information per spike [51]

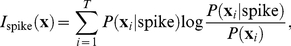
(6)where 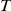 is the total number of stimuli. The mutual information between the stimulus features and the neural response (the presence of a spike, 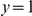, or its absence, 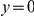) is a sum of contributions from both types of responses: 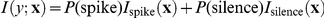, with 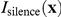 defined by replacing 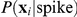 with 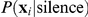 in Eq. (6). However, in the limit of small time bins where 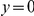 in most of the bins, 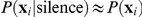, which leads to vanishing contributions from 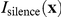. In this case, one can optimize either 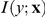 or 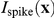 to find the relevant features 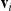 along which the probability distribution 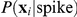 is most different from 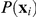 according to the Kullback-Leibler distance, cf. Eq. (6). We note that this optimization is not convex and therefore a standard gradient ascent algorithm may not find the global maximum. An algorithm that combines stochastic gradient ascent with simulated annealing is publicly available at http://cnl-t.salk.edu.

To extend the MID algorithm to nonlinear MID (nMID), the stimulus is simply transformed by a nonlinear operation. For the second order nonlinear transformation considered in this paper, 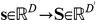, where 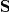 is a vector whose first 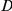 components are the components of 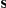 and the remaining components are the elements of 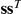. Due to the symmetry of the outer product matrix, this transformed stimulus dimensionality is 
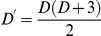
. In this new space, the MID algorithm works as before, finding a linear combination of these dimensions, i.e. 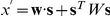, such that 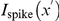 is maximized. To improve performance and cut down on runtime, the search was started from the minimal model estimate 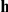 for 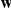 and 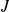 for 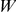.

To prevent overfitting of the parameters, an early stopping mechanism was used whereby the data was broken into two sets: one set was used for training and the other used for testing. The training set was used to search the parameter space, while the test set was used to evaluate the parameters on independent data. The best linear combination for both data sets was returned by the algorithm. This procedure was done four times, using four different quarters of the complete data set as the test set. The resulting parameters found from these four fittings were averaged before diagonalizing and finding the relevant features. Unlike the regularization of STC models, this procedure can be used when analyzing experimental data.

### Minimal models

The model of the neural response that matches experimental observations in terms of the mean response probability, as well as correlations between the neural response with linear and quadratic moments of stimuli can be obtained by enforcing
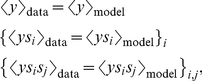
(7)where 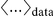 is an average over 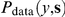 and 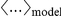 is an average over 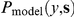. Because 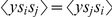, this reduces to a set of
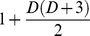
(8)equations. Simultaneously satisfying these equations is analytically equivalent to maximizing the log likelihood of the data [49], which is convex and can therefore be maximized using a conjugate gradient ascent algorithm.

To prevent overfitting of the parameters, an early stopping procedure was implemented similar to that used in the MID algorithm. Each step of the algorithm increased the likelihood of the training set, but at some point began decreasing the likelihood of the test set, indicating the fitting of noise within the training set. The algorithm then returned the parameters found at the maximum likelihood of the test set. As described above, this was done four times with different quarters of the data serving as the test set and the resulting parameter vectors were averaged before diagonalizing the matrix 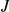.

Significance testing of the eigenvalues was done by creating 500 Gaussian distributed random matrices with the same variance as that of the set of elements of 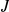. These random matrices were each diagonalized to create a random eigenvalue distribution. Eigenvalues of 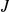 were then said to be significant if they fell below the lower 2.5^th^ percentile or above the 97.5^th^ percentile.

### Physiology experiments

The data analyzed in this paper were collected in a previous study [48] and the details are found therein. The stimulus was a spot of light covering a cell's receptive field center, flickering with non-Gaussian statistics that mimic those of light intensity fluctuations found in natural environments [30], [38]. The values of light intensities were updated every 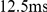 (update rate 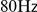). The spikes were recorded extracellularly in the LGN with high signal-to-noise ratio, allowing for excitatory post-synaptic potentials generated by the RGC inputs to be recorded. From such data, the complete spike trains of the RGCs could be reconstructed.
